# Performance Enhancement of PLA-Based Blend Microneedle Arrays through Shish-Kebab Structuring Strategy in Microinjection Molding

**DOI:** 10.3390/polym15102234

**Published:** 2023-05-09

**Authors:** Lifan Zhang, Yinghong Chen, Jiayu Tan, Shuo Feng, Yeping Xie, Li Li

**Affiliations:** The State Key Laboratory of Polymer Materials Engineering, Polymer Research Institute of Sichuan University, Chengdu 610065, China; zhanglifan1998@163.com (L.Z.);

**Keywords:** polylactic acid, blend, microneedle, microinjection molding, shish-kebab structure

## Abstract

Poly(lactic acid) (PLA) microneedles have been explored extensively, but the current regular fabrication strategy, such as thermoforming, is inefficient and poorly conformable. In addition, PLA needs to be modified as the application of microneedle arrays made of pure PLA is limited because of their easy tip fracture and poor skin adhesion. For this purpose, in this article, we reported a facile and scalable strategy to fabricate the microneedle arrays of the blend of PLA matrix and poly(p-dioxanone) (PPDO) dispersed phase with complementary mechanical properties through microinjection molding technology. The results showed that the PPDO dispersed phase could be in situ fibrillated under the effect of the strong shear stress field generated in micro-injection molding. These in situ fibrillated PPDO dispersed phases could hence induce the formation of the shish-kebab structures in the PLA matrix. Particularly for PLA/PPDO (90/10) blend, there are the densest and most perfect shish-kebab structures formed. The above microscopic structure evolution could be also advantageous to the enhancement in the mechanical properties of microparts of PLA/PPDO blend (tensile microparts and microneedle arrays), e.g., the elongation at break of the blend is almost double that of pure PLA while still maintaining the high stiffness (Young’s modulus of 2.7 GPa) and the high strength (tensile strength of 68.3 MPa) in the tensile test, and relative to pure PLA, there is 100% or more increase in the load and displacement of microneedle in the compression test. This could open up new spaces for expanding the industrial application of the fabricated microneedle arrays.

## 1. Introduction

As the demand for miniaturization and refinement of polymer applications is increasing, high-precision processing methods witness a rapid development. The thriving advancement of micro/nanoprocessing technologies, such as micro/nanomolding, high-energy beam processing and micro/nanomachining, promotes the development of multifunctional device fabrication [1,2,3]. Microinjection molding has gained much attention among these processing methods due to its excellent molding accuracy and high processing efficiency [4,5], thus making it easy to be industrialized. Many polymer components and devices applied in the medical field, such as transnasal inhalers, vascular clips, bone screws and microneedles [6,7,8], generally require the precision manufacturing (good replication accuracy) and processing efficiency so as to achieve good economic benefits. Obviously, the microinjection molding strategy could exhibit significant advantages in the manufacturing of micro medical devices. In these medical microparts, the microneedle arrays are well known as a new ideal mode of transdermal drug delivery which has the advantages of painlessness, non-invasiveness, and high drug delivery efficiency [9,10,11]. According to the way of drug delivery, the microneedle arrays could be classified into solid microneedles, coated microneedles, soluble microneedles, hollow microneedles and so on [12]. Moreover, the coated microneedles have received increasing attention from researchers in recent years for their wide application, but the underlying microneedle substrates are still largely prepared through thermoforming method [13,14,15] with low production efficiency, poor precision, and complex processing procedures, making them difficult to be extensively applied in the medical field. In order to overcome the above challenge, here the microinjection molding technology was applied to fabricate the microneedle substrates with good comprehensive properties for coating.

Regarding the selection of materials for the preparation of microneedles, careful considerations should be conducted. Indeed, PLA is the most cost-effective biodegradable material with good biocompatibility, complete degradability and good mechanical properties [16,17]. It seems to be the most widely used material for the fabrication of microneedle substrates, also for the reason that the outstanding features of PLA could meet the basic requirements of microneedles for the high material strength in penetrating the skin [18,19,20]. However, the high rigidity and poor toughness of pure PLA may lead to needle tip breakage and non-adherence of the substrate to the skin during the microneedle administration [21]. Meanwhile, there are two major factors which should be considered in selecting materials for medical manufacturing. The first one is good material biocompatibility, especially in microneedle applications where these materials are designed to penetrate the skin’s stratum corneum; the second one is the complete material biodegradability and simple formulation composition, which can facilitate recycling and cost control. On the basis of the above considerations, the construction of shish-kebab crystalline structures [22] could be one of the effective toughening strategies for PLA through the addition of a limited number of other biodegradable components. Formation of such shish-kebab structures could generally be induced by the incorporation of the proper amount of poorly compatible dispersed phases in the matrix under the effect of the strong shear stress field [23]. It is noted that poly(p-dioxanone) (PPDO), a biopolymer with both ester and ether bonds in the main chain, exhibits excellent flexibility, high tensile strength and good biocompatibility and is used widely in the medical field [24]. Therefore, here we attempt to achieve the balance between the microneedle tip stiffness and the substrate flexibility by blending PLA and PPDO which are two incompatible polymers with complementary mechanical properties.

In order to realize the creation of the shish-kebab crystalline structures in the PLA/PPDO blend system, it is first necessary to make PPDO uniformly dispersed in the PLA matrix. Then, taking advantage of the strong shear force field during processing, the PPDO dispersed phases could be in situ fibrillated along the flow direction to form the highly oriented fibrillar structure [6,25] which could possibly act as shish during crystallization and then be advantageous to the shish-kebab crystallization formation. As we know, extrusion and injection molding are the two well-known polymer processing techniques featured with shear force field. Moreover, compared with conventional injection molding, the most striking feature of the microinjection molding technology is its temperature gradient and powerful shear stress field due to its small runner/cavity size effect [7,26,27] which could be highly beneficial for the formation of shish-kebab structures. However, because of the poor compatibility between PPDO and PLA, the excessive surface area of the PPDO dispersed phase, which is resulted from the PPDO content increase, may lead to slippage at the phase interface under the effect of the shear force, thus negatively affecting the formation of in situ fibers and its toughening effect. As a result, the effect of PPDO content on the formation of shish-kebab crystallization structure in PLA/PPDO blend systems under microinjection molding conditions is worth investigating.

Accordingly, in this paper, the influence of PPDO content on the crystallization and mechanical properties of PPDO/PLA blend micropart was fully investigated. The incorporated PPDO particles as dispersed phase proved to be able to promote PLA/PPDO blend to form the shish-kebab structures which could effectively enhance the blend toughness (increased by more than 70% for elongation at break) while maintaining the high stiffness (Young’s modulus of 2.7 GPa) and strength (tensile strength of 68.3 MPa) of PLA. In addition, the microneedles prepared by microinjection molding also showed a similar increase tendency in compression load. As a result, the fabricated PPDO/PLA microneedles could possess good application prospects as the substrates of the coated microneedles.

## 2. Materials and Methods

### 2.1. Material

The raw material of poly (lactic acid) (PLA, 4032D) with a weight-average molecular weight (Mw) of about 100 kDa was bought from UNIC Technology Co., Ltd. (Suzhou, China). The above PLA has a melting flow index of 3.87 g/min (190 °C, 2.16 kg load) and a melting temperature of about 170 °C. The poly(p-dioxanone) (PPDO) with a melting temperature of about 109 °C and intrinsic viscosity of 2 dL/g was supplied by Shenzhen Polymtec Co. (Shenzhen, China).

### 2.2. Preparation of PLA/PPDO Blend Microneedle and Micro Tensile Sample

**Figure 1:**
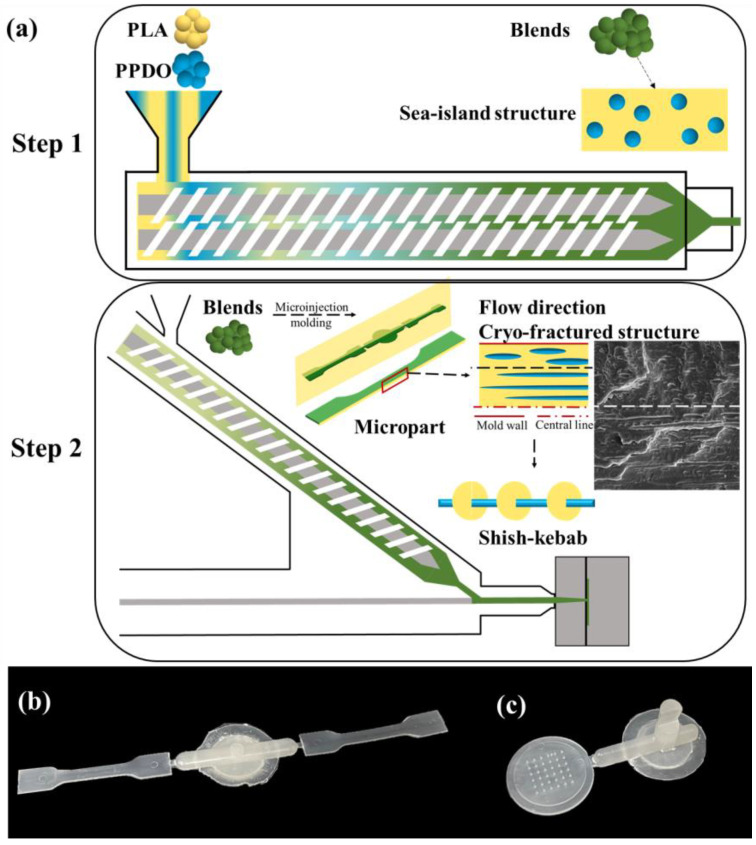
The schematic diagram for preparation of the PLLA/PPDO blend microparts (**a**), the digital picture of the micro tesile sample (**b**) and the microneedle array (**c**).

During the processing of PLA, the adsorbed moisture can cause the degradation of PLA and, hence, the performance deterioration. To avoid this phenomenon, the PLA and PPDO pellets were priorly dried at 80 and 40 °C in the vacuum for 24 h, respectively. The related preparation processes are illustrated in Figure 1. The well-mixed mixture of the dried PLLA and PPDO was efficiently melt-compounded in a twin-screw extruder (ϕ = 25 mm, L/D = 40, LZ-80/VS, Labtech Engineering Co., Ltd.) in the temperature range of 160−180 °C. The screw speed was held at 80 r/min so as to reduce melt residence time in the extruder, mitigate thermal degradation and properly maintain a certain shear action on the blends which is beneficial for the formation of PPDO nanodroplets. The weight fraction content of PPDO in blend we explored is 0, 5, 10 and 15%. Then, the obtained blend extrudates were cooled and pelletized. The dried pellets were subsequently injection-molded into micro tensile parts and microneedle arrays using a Battenfeld MicroPower 5 microinjection molding machine (GmbH, Kottingbrunn, Austria). The microinjection molding conditions we used include the injection speed of 100 mm/s, mold temperature of 40 °C, injection pressure of 1500 bar and melt temperature of 180 °C. For convenience, the melt-compounded PLLA/ PPDO blends (by extrusion) with different PPDO loading are denoted as b-x, where x represents the weight fraction of PPDO in the blend, for example, b-10 means that there are 10 wt % PPDO-dispersed phases in the PLA/PPDO blend. Similarly, the corresponding microinjection molded samples are denoted as the m-x (m-0, m-5, m-10 and m-15); the microneedle arrays are denoted as the n-x (n-0, n-5, n-10 and n-15).

## 3. Characterizations

### 3.1. Differential Scanning Calorimetry (DSC)

The Q20 (TA company, Newcastle, DE, USA) differential scanning calorimeter was applied to investigate the crystallization and melting behavior of PLA/PPDO blend micropart. About 6–9 mg samples sealed in an aluminum crucible were firstly cooled from 40 °C to 0 °C at a cooling rate of 10 °C/min and then heated to 200 °C at a heating rate of 10 °C/min under a nitrogen atmosphere with a flow rate of 50 mL/min. The PLA crystallinity degree in the blend was calculated using the following formula:

(1) $$X_{c,PLA}\left(\%\right)=\frac{{\Delta H}_{m.PLA}-{\Delta H}_{c.PLA}}{\Delta H_{0.PLA}\omega_{PLA}}\times 100\%$$

where $\omega_{PLA}$ is the weight fraction of PLA in the blend; ${\Delta H}_{m.PLA}$ is the melting enthalpy of PLA; ${\Delta H}_{c.PLA}$ is the PLA cold crystallization enthalpy; $\Delta H_{0.PLA}$ is the full PLA crystallization enthalpy (93.0 J/g) [28].

### 3.2. Scanning Electronic Microscopy (SEM)

The blend filament or microinjection molded samples were completely submerged in liquid nitrogen for 10 min and cryo-fractured in the flow direction. The amorphous PLA is removed for clearer visualization of the crystal structure in the microinjection molded samples. The cryo-fractured samples were etched in the 0.05 mol/L NaOH methanol solution at 25 °C for 24 h. The etched samples were ultrasonically cleaned in distilled water for 5 min to remove surface impurities. Finally, a thin gold layer was deposited on the fractured sections of all samples by vacuum spraying. The microscopic morphology of the samples was observed at 5 kV accelerating voltage with JSM-5900LV SEM (JEOL, Akishima, Japan). Nano Measure software was used to count the number, dimension and dimensional distribution of dispersed phases in the SEM photographs.

### 3.3. Two-Dimensional Small-Angle X-ray Scattering (2D-SAXS)

The two-dimensional small-angle X-ray scattering (2D-SAXS) measurements were carried out on Xeuss 2.0 SAXS equipment (Xenocs, Grenoble, France), and the scattering signals were recorded with a Pilatus 300 K detector of Dectris (Baden-Dättwil, Switzerland).

### 3.4. Mechanical Property Measurement

The tensile tests of PLA/PPDO blend micro tesile samples were carried out on an Instron 5567 tester (INSTRON company, Norwood, MA, USA) with a maximum loading of 1 kN and a cross-head speed of 1 mm/min.

The compression experiments of the microneedle arrays were conducted on an Electro-Force 3220 SERIES II testing machine (Bose company, Framingham, MA, USA) with supporting software WinTest7.

## 4. Results and Discussion

### 4.1. Phase Morphology Analysis

**Figure 2:**
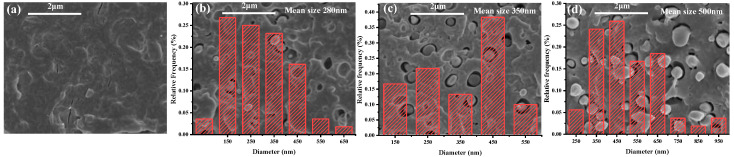
SEM images of the cryo-fractured surfaces and the corresponding particle size statistics of b-0 (**a**), b-5 (**b**), b-10 (**c**) and b-15 (**d**).

**Figure 3:**
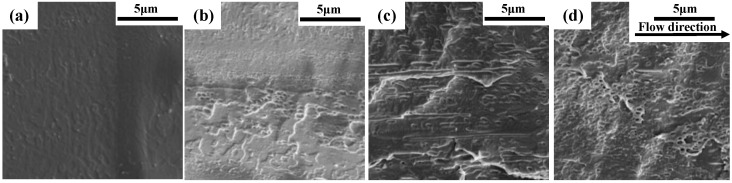
SEM images of the cryo-fractured surfaces of m-0 (**a**), m-5 (**b**), m-10 (**c**) and m-15 (**d**).

PLA (as polymer matrix) and PPDO (as dispersed phase) are two biodegradable and biocompatible polymers which are suitable for the fabrication of microneedles. However, they are incompatible in nature and under the effect of the very strong shear force field generated in microinjection molding (the shear rate can achieve as high as 10^6^ s^−1^ [29]), the PPDO dispersed phase can be expected to be in situ fibrillated and then provokes the PLA crystallization to form the shish-kebab structures. It is very interesting to investigate the morphological evolution of the PLA/PPDO blend under the microinjection molding conditions. Figure 2 shows the morphologies of the microinjection molded PLA/PPDO blend with different PPDO contents. It can be seen that there are relatively large voids existing between the PPDO dispersed phase and the PLA matrix, and this indicates that PPDO is really incompatible with PLA. Under the microinjection molding condition, the particle size of PPDO is almost near the nanoscale, for example, the average size of the PPDO dispersed phase in the PLA/PPDO blend with 5, 10 and 15 wt% of PPDO content is found to be 280, 350 and 500 nm, respectively, which are much smaller than that of the regular immiscible blend system [28,30]. In addition, the increase in PPDO content results in an increase in the domain size of PPDO dispersed phase (from 280 nm to 500 nm). It is also noted that, with increasing PPDO content, the number of PPDO dispersed phase particles presents an increasing tendency. The reason for this is that with increasing PPDO content, there would be more amount of PPDO droplets formed and stretched under the effect of the shear stress field in microinjection molding conditions. Due to the high cooling temperature gradient, the stretched PPDO droplets would be instantly solidified. Upon increasing the PPDO content, it is possible that the PPDO droplets with size increase are produced. Furthermore, from Figure 2, it is also seen that the most homogeneous particle size distribution of the PPDO dispersed phase is found in the b-10 sample, and the size difference between different dispersed phase particles is the largest in the b-15 sample. This means that the PPDO agglomeration would occur when the PPDO content reaches 15% or more. Figure 3 shows the SEM image of the PLA/PPDO blend micro tensile sample cryo-fractured along the melt flow direction. As can be seen, there are clearly the fibrillar structures of the PPDO dispersed phase generated in the PLA matrix which are resulted from the stretching of the PPDO dispersed phase droplets under the very strong shear stress field of microinjection molding and the subsequent rapid cooling process. In addition, there are hierarchical structures existing in the cross-section of PLA/PPDO blend micropart perpendicular to the melt flow direction. It is known that in the frozen core layer near the cavity center, the shear stress field exerting on the melt is relatively weaker and the resultant stretching deformation would be smaller. As a result, the sheared spherical melt droplets of the PPDO dispersed phase would be slightly stretched to produce spindle-shape particles with a relatively low aspect ratio; however, in the shear layer away from the cavity center as the melt droplets of the PPDO dispersed phase suffer from the substantially enhanced shear stress field, they are heavily stretched into the submicron fibrillar structures with a relatively high aspect ratio along the melt flow direction.

### 4.2. Crystallization Behavior Analysis

**Figure 4:**
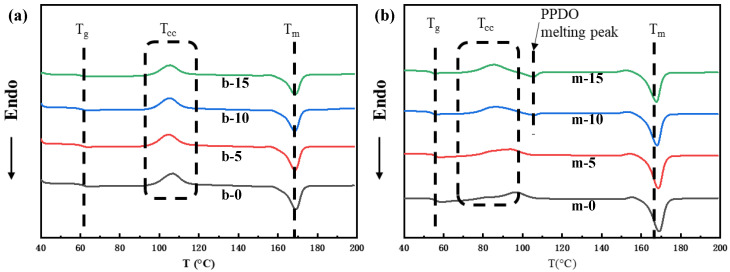
The DSC heating curves of PLA/PPDO blend extrudate pellets (**a**) and microinjection molded tensile parts (**b**) with different PPDO content.

DSC measurement was used to investigate the crystallization melting behavior of the PLA/PPDO blend with different PPDO contents (melt-compounded sample and microinjection molded sample). The obtained DSC curves are shown in Figure 4 and the corresponding parameters are included in Table 1. As can be seen, in the DSC heating curve of the PLA/PPDO blend extrudate sample (Figure 4a), there are three peaks clearly distinguished, including the PLA enthalpy relaxation peak around 63 °C (also in the glass transition region), the PLA cold crystallization peak around 105 °C and the PLA melting peak around 170 °C. The influence of PPDO content on the thermal properties of the PLA/PPDO blend is not much, e.g., the crystallinity of PLA increases slightly as the PPDO content increases (the crystallinity only increases by 1.56% upon the increase of PPDO loading from 0 to 15%). Comparatively, for the microinjection molded PLA/PPDO blend sample (Figure 4b), there are four peaks clearly distinguished, including the melting peak of PPDO newly appearing around 107 °C (when PPDO content increases to 10~15%) except for the three peaks appearing in Figure 4a. It is interestingly noted that the crystallization behavior of the microinjection molded PLA/PPDO blend micropart (Figure 4a) is obviously different from that of the conventionally melt-compounded PLA/PPDO blend (Figure 4b). Firstly, compared with the melt-compounded PLA/PPDO blend, the glass transition temperature (T_g_), the cold crystallization temperature (T_cc_) and the cold crystallization melting enthalpy (∆H_m_) of PLA in the microinjection molded samples are significantly decreased. In addition, with increasing PPDO content, the T_cc_ and T_g_ of PLA both present a decreasing tendency. The reason for the decrease in T_g_ of PLA could be related to the formation of the oriented structure in the microinjection molded PLA/PPDO blend. Under microinjection molding conditions, the oriented molecular chains are instantly frozen due to the high cooling temperature gradient. As a result, there is unidirectional tension stress in the frozen-oriented molecular chains of PLA. Under the effect of such tension stress, the glass transition of PLA could occur at a lower temperature, indicating a decrease in T_g_. Of course, the high flexibility of PPDO molecular chains would lead to a further decrease in T_g_ under microinjection molding conditions. In addition, the T_cc_ decrease above mentioned could be explained by the fact that microinjection molding and incorporation of PPDO can enhance the crystallization capability of PLA. The appearance of the PPDO melting peak indicates that microinjection molding processing can also improve the crystallization capability of PPDO. Particularly, it should be stressed that the PLA T_cc_ decreases with PPDO content increasing is attributed to the flexible chain segment of PPDO accelerating the crystallization of PLA. The above results are again verified by the change in crystallinity of PLA. As shown in Table 1, the crystallinity of PLA in the microinjection molded sample is almost double as high as that in the melt-compounded sample. It is also noted that the incorporation of PPDO and the PPDO content increase equally resulting in the increase of PLA crystallinity to a certain degree. This suggests that the strong shear stress field present in microinjection molding processing can really promote the orientation of PLA macromolecular chains and further help them to form the crystal lattices, thus favoring the crystallization of PLA, i.e., shear-induced crystallization. On the other hand, the high flexibility of PPDO molecular chains can also truly improve the crystallization of PLA under microinjection molding conditions.

**Figure 5:**
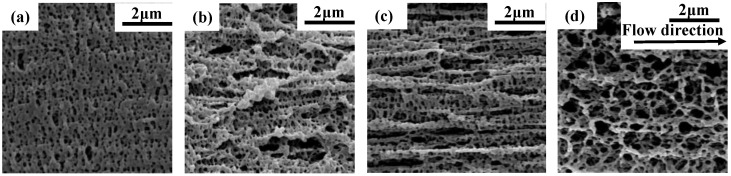
The cryo-fractured surfaces of PLA/PPDO blend micro tensile parts of m-0 (**a**), m-5 (**b**), m-10 (**c**) and m-15 (**d**) after etching treatment along melt flow direction.

Figure 5 shows the SEM crystallization morphology of microinjection molded PLA/PPDO blend micro tensile sample cryo-fractured along the melt flow direction at different PPDO content. Before observation, the fractured surface of the PLA/PPDO blend micropart was etched by 0.05 mol/L NaOH methanol solution. It can be seen that, very interestingly, for pure PLA (Figure 5a), there are only the lateral lamellae (kabab) crystal structures formed after etching the pure PLA micropart. However, for the PLA/PPDO blend micropart, there are clearly well-defined shish-kebab orientation structures formed along the melt flow direction as shown in Figure 5b–d. It is clear that, with increasing PPDO content to 10%, there are more perfect and more shish-kebab structures generated; however, when the PPDO content further increases to 15%, both the number and the perfection degree of the formed shish-kebab structures are obviously reduced. The above results show that only an appropriate content of PPDO dispersed phase (10%) is beneficial for the formation of the shish-kebab structures. The shish structures consist of submicron fibrillae of the PPDO dispersed phase, while the kebab structures consist of PLA lamellae. The formation mechanism is based on the shear-induced crystallization in microinjection molding (very strong shear stress field existing). The influence of PPDO content on formation of the shish-kebab structures could be related to formation of the fibrillae structures of the PPDO dispersed phase. At the higher PPDO content, there are the formed PPDO fibers with bigger radial size and the agglomeration of PPDO dispersed phase would occur which is not advantageous to the formation of shish-kebab structures. This can be further verified by the following SAXS characterization.

**Figure 6:**
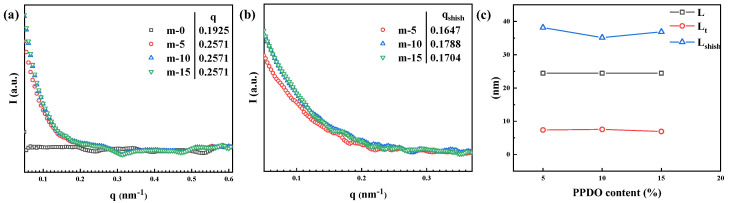
The 1D-SAXS intensity profiles of long period integration curve (**a**), shish signal integration curve (**b**), calculated long period (L) (**c**), average lamellar crystal thickness (L_t_), shish fibrilla spacing (L_shish_) and kebab lateral size (L_lateral_) at different PPDO content.

The formed shish-kebab structures were further characterized using the SAXS measurement. The results are shown in Figure 6. As can be seen, the shish signal in the equatorial direction can be clearly reflected in the 1D-SAXS profiles (Figure 6b). In the shish-kebab structure, the kebab crystals grow around the shish, thus defining the long period (L) of the kebab crystals and their adjacent amorphous regions [31]. As a result, the average thickness of the kebab lamellar crystal (L_t_) can be calculated from the long period and the crystallinity [32]:

(2) $$L=\frac{2\pi }{q}$$

(3) $$L_{t}=L\times X_{c}$$

After calculation, the addition of PPDO could lead to a decrease in the average thickness of lamellar crystals of PLA. Integrating the shish signal in the 1D-SAXS intensity profile, the fiber spacing (L_shish_) can be calculated from the position of the curve where the shoulder peak appears (q_shish_) [33]:

(4) $$L_{\mathrm{shish}}=\frac{2\pi }{q_{\mathrm{shish}}}$$

After calculation, the L_shish_ parameters of m-5, m-10 and m-15 microparts achieve 38.15 nm, 35.14 nm and 36.87 nm, respectively. As can be seen, with the increase of PPDO content, the Lt of PLA first increases and then decreases, and the L_shish_ first decreases and then increases. The maximum Lt and the minimum L_shish_ appear at 10% PPDO content. The reason for this is that as PPDO content increases, the PPDO dispersed phase could form a greater number of fibrilla structures of shish under the effect of the shear stress field. As a result, the fiber spacing decreases, and the formation of PLA kebab lamellar crystals could be promoted. With further increase of PPDO content, there is an agglomeration of the PPDO dispersed phase occurring, leading to an increase in the voids in the PLA matrix and hence the increase in PPDO fiber spacing. Correspondingly, the PLA phase that can be crystallized around PPDO fiber would decrease, thus resulting in a decrease in the thickness of lamellar crystals.

### 4.3. Mechanical Property Measurement

**Figure 7:**
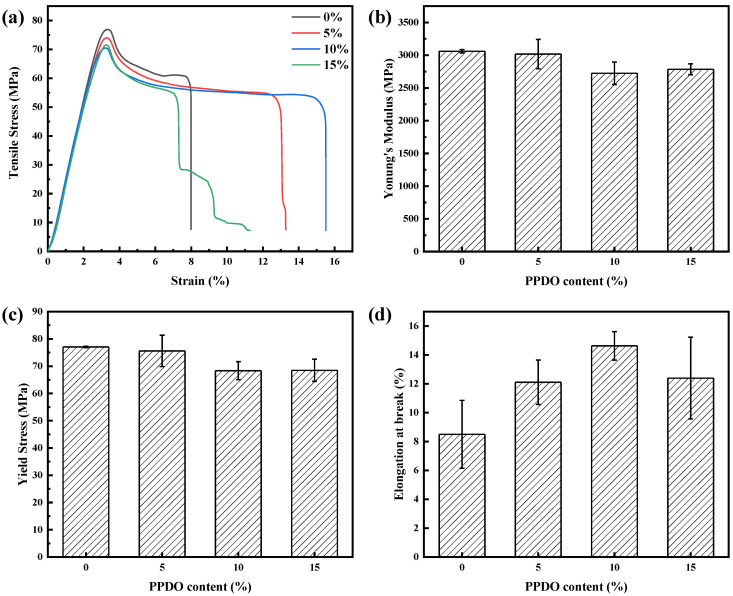
The stress-strain curves of the PLA/PPDO blend microparts with different PPDO content (**a**), and the corresponding Young’s modulus (**b**), yield strength (**c**) and elongation at break (**d**).

Figure 7 shows the effect of PPDO content on the mechanical properties of the microinjection molded samples. The stress–strain curves of the PLA/PPDO blend micro tensile samples with different PPDO content at a crosshead speed of 1 mm/s are exhibited in Figure 7a. It can be seen that, with increasing PPDO content, the tensile strength and Young’s modulus of the PLA/PPDO blend micropart present a decreasing tendency due to the existence of the flexible PPDO dispersed phase and the incompatibility between both phases (the tensile strength and Young’s modulus remain a relatively high value). However, the decrease degree is not much (the tensile strength of all samples is in the range of 68~77 MPa and Young’s modulus is in the range of 2.8~3.1 GPa). This could be attributed to the formation of shish-kebab crystallization structures. Comparatively, the change in the elongation at break with the PPDO content is different from that in both tensile strength and Young’s modulus. With increasing the PPDO content, the elongation at break shows a first increasing and then decreasing tendency and the highest value (15.8%) appears at 10% PPDO content, where the elongation at break is almost double that of pure PLA. Actually, the changing tendency of elongation at break is similar to that of the shish-kebab structure formation with the PPDO content. The above results show that the formation of a shish-kebab structure in PLA/PPDO blend micropart is advantageous to the enhancement of the corresponding elongation at break, i.e., the toughness of PLA/PPDO blend micropart which is the basis for fabrication of the high-performance PLA/PPDO blend microneedle.

**Figure 8:**
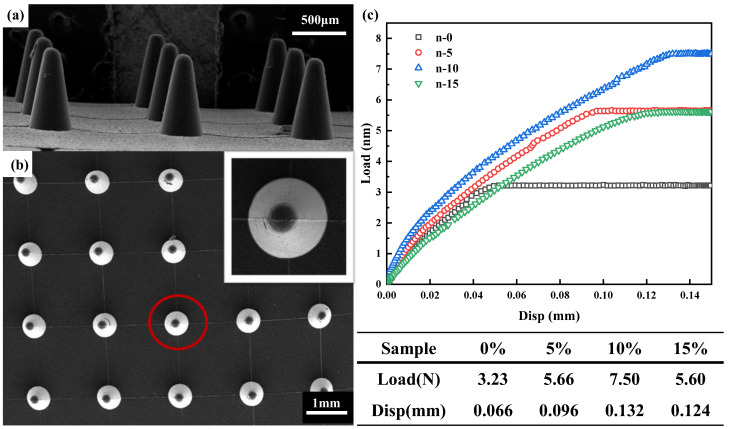
The SEM morphology of the microinjection molded PPDO/PLA blend microneedles of side view (**a**) and top view (**b**), and the inset shows a magnification of one microneedle marked by the red circle; the compression load–displacement curves of the PPDO/PLA blend microneedles with different PPDO content (**c**).

On the basis of the above investigation and results, the microneedle arrays of PLA/PPDO blend with different PPDO content were successfully prepared through microinjection molding technology. The SEM images of the prepared microneedle arrays are shown in Figure 8, where a and b exhibit the side view and top view of the microneedle arrays, respectively. It can be seen that there is a very good replication property for the prepared microneedle arrays which possess a well-defined profile, excellent conformability and smooth surface. The dimensional parameters of the fabricated microneedle arrays could include: the diameter of the microneedle substrate is 12 mm, and the substrate thickness is 0.9 mm; the bottom diameter of each microneedle is 0.3 mm, and the height is 0.6 mm. All these microneedles are distributed in a 6 × 6 array. In order to investigate the toughness improvement of the microneedle tip (so that it is not easy to break the microneedle tip upon working in the skin), the microinjection molded microneedle samples of n-0, n-5, n-10 and n-15 were subjected to the fracture experiments. The corresponding results are shown in Figure 8c. It can be seen that the optimum mechanical performance of the fabricated PLA/PPDO blend microneedle appears at 10% PPDO content. The maximum fracture load is 7.5 N and the maximum displacement is 0.132 mm. Comparatively, for the microneedle prepared at 5% and 15% PPDO, the corresponding fracture load achieves 5.66 N and 5.6 N, respectively, while the displacement reaches 0.096 mm and 0.124 mm, respectively. The above results show that the incorporated PPDO dispersed phase has a toughening effect on the tip of the microneedle array which could greatly increase its fracture load and displacement. As discussed before, the toughening effect should be attributed to the formation and construction of the shish-kebab crystallization structures in PLA/PPDO blend systems under microinjection molding conditions.

## 5. Conclusions

In summary, in this study, the PLA-based microneedles with enhanced mechanical performance were successfully fabricated by the formation and construction of the shish-kebab structures by combining the incorporation of a small amount of PPDO and microinjection molding processing strategies. The enhanced toughness of the PLA/PPDO blend microneedle array system was attributed to the successful construction of the shish-kebab structures, where the shish consists of the formed submicron fibrilla structure of PPDO dispersed phase and the kabab consists of the formed PLA lateral lamellae structure under the effect of strong shear stress field generated during microinjection molding. The PPDO content exhibits an important influence on the formation of shish-kebab crystallization structures, e.g., the most perfect and the greatest number of shish-kebab structures could be formed when the PPDO content is at 10%. Correspondingly, at this PPDO concentration, the average particle size of the PPDO dispersed phase is at 350 nm. The formation of the shish-kebab structures could be also reflected in the enhancement in the mechanical properties of PLA/PPDO blend micropart, i.e., the tensile property of the tensile micropart and the compression property of the microneedle arrays. The elongation at break of PLA/PPDO (10%) blend is almost double that of pure PLA, and the compression load and displacement of PLA/PPDO (10%) blend microneedle was found to increase by 132% and 100%, respectively, relative to pure PLA material. It could be envisioned that the high-performance PLA/PPDO blend microneedle arrays we fabricated will find their expected broad and good application prospects in the field of transdermal drug delivery.

## Figures and Tables

**Table 1:** The DSC parameters of the PLA/PPDO blend extrudate pellets and microparts with different PPDO content.

Sample	T_g-PLA_ (°C)	T_cc-PLA_ (°C)	ΔH_cc-PLA_ (J/g)	T_m-PPDO_ (°C)	T_m-PLA_ (°C)	ΔH_m-PLA_ (J/g)	X_c-PLA_ (%)
b-0	63.27	105.21	22.43	/	170.04	34.20	12.66
b-5	62.60	104.90	22.18	/	169.66	33.56	12.88
b-10	62.70	105.64	18.78	/	170.20	29.91	13.30
b-15	62.20	105.36	20.44	/	169.58	31.68	14.22
m-0	55.05	99.32	14.79	/	169.31	39.76	26.85
m-5	53.49	95.89	13.29	/	168.64	39.97	30.20
m-10	53.30	91.32	14.16	107.54	169.93	40.08	30.97
m-15	52.08	90.94	13.82	107.70	168.42	36.24	28.36

Note: T_g_, T_cc_, ΔH_cc_, T_m_ and ΔH_m_ are the glass transition temperature, cold crystallization temperature, cold crystallization melting enthalpy, melting temperature and melting enthalpy, respectively.

## Data Availability

Not applicable.
